# Intervention fidelity in the definitive cluster randomised controlled trial of the Healthy Lifestyles Programme (HeLP) trial: findings from the process evaluation

**DOI:** 10.1186/s12966-017-0616-6

**Published:** 2017-11-28

**Authors:** Jenny Lloyd, Sarah Dean, Siobhan Creanor, Charles Abraham, Melvyn Hillsdon, Emma Ryan, Katrina M. Wyatt

**Affiliations:** 1 Institute of Health Services Research, University of Exeter Medical School (formerly Peninsula College of Medicine and Dentistry), South Cloisters, St Luke’s Campus, Exeter, Devon EX1 2LU UK; 2 Peninsula Clinical Trials Unit and Medical Statistics, Plymouth University Peninsula Schools of Medicine & Dentistry (formerly Peninsula College of Medicine and Dentistry), ITTC Building, Plymouth Science Park, Plymouth, Devon PL6 8BX UK; 3 Sport and Health Sciences, College of Life and Environmental Sciences, University of Exeter, St Luke’s Campus, Heavitree Road, Exeter, Devon EX1 2LU UK; 4 Isca Academy, Earl Richards Road, Exeter, Devon EX2 6AP UK

**Keywords:** Cluster RCT, School-based intervention, Process evaluation, Fidelity, Programme integrity, Engagement, Obesity, Mixed methods, Research waste

## Abstract

**Background:**

The Healthy Lifestyles Programme (HeLP) was a novel school-located intervention for 9–10 year olds, designed to prevent obesity by changing patterns of child behaviour through the creation of supportive school and home environments using dynamic and creative delivery methods. This paper reports on both the quantitative and qualitative data regarding the implementation of the HeLP intervention in the definitive cluster randomised controlled trial, which was part of the wider process evaluation.

**Methods:**

Mixed methods were used to collect data on intervention uptake, fidelity of delivery in terms of content and quality of delivery of the intervention, as well as school and child engagement with the programme. Data were collected using registers of attendance, observations and checklists, field notes, focus groups with children and semi-structured interviews with teachers. Qualitative data were analysed thematically and quantitative data were summarized using descriptive statistics.

**Results:**

All 16 intervention schools received a complete or near complete programme (94–100%), which was delivered in the spirit in which it had been designed. Of the 676 children in the intervention schools, over 90% of children participated in each phase of HeLP; 92% of children across the socio-economic spectrum were deemed to be engaged with HeLP and qualitative data revealed a high level of enjoyment by all children, particularly to the interactive drama workshops. Further evidence of child engagment with the programme was demonstrated by children’s clear understanding of programme messages around marketing, moderation and food labelling. Thirteen of the intervention schools were deemed to be fully engaged with HeLP and qualitative data revealed a high level of teacher ‘buy in’, due to the programme’s compatability with the National Curriculum, level of teacher support and use of innovative and creative delivery methods by external drama practitioners.

**Conclusion:**

Our trial shows that it is possible to successfully scale up complex school-based interventions, engage schools and children across the socio-economic spectrum and deliver an intervention as designed. As programme integrity was maintained throughout the HeLP trial, across all intervention schools, we can be confident that the trial findings are a true reflection of the effectiveness of the intervention, enabling policy recommendations to be made.

**Trial registration:**

ISRCTN15811706.

**Electronic supplementary material:**

The online version of this article (10.1186/s12966-017-0616-6) contains supplementary material, which is available to authorized users.

## Background

Obesity is considered to be one of the greatest challenges facing public health in the twenty-first Century. In England, currently a fifth of boys and girls start school overweight or obese and one third of children leave primary school (aged 11 years old) overweight or obese [1]. Childhood obesity is strongly associated with socioeconomic status, with children from the least affluent decile being twice as likely to be obese as children from the most affluent decile [2]. Obese children are five times more likely to be obese as adults than non-obese children and 80% of obese adolescents were still obese in adulthood [3].

School-based obesity preventative interventions have the potential to reach a large number of children and families across the socioeconomic spectrum and schools provide the organisational, social and communication structures to educate children and parents about healthy lifestyles. Systematic reviews of school-based interventions to prevent obesity and/or increase physical activity and reduce sedentary behaviours show, at best, moderate evidence of effectiveness, with the majority of studies being conducted in the United States [4]. Several methodological short-comings with the studies have been identified such as differential loss to follow up, not having sufficient statistical power to detect clinically meaningful differences between groups and short term follow up [5, 6]. In addition, programme integrity is rarely reported in primary and early secondary prevention programmes in general [7] and those trials which do report on the fidelity of delivery, often reveal a lack of intervention integrity, calling into question the validity of their findings [8, 9].

The verification of programme integrity, also known as *intervention fidelity*, (i.e. the extent to which the intervention was delivered as it was designed or written to be delivered) is of particular importance. Without such verification, distinguishing between outcomes that may be attributed to a lack of fidelity to delivery and those outcomes attributed to the inability of the intervention to achieve the desired result is extremely difficult. Failure to deliver an intervention as intended can lead to unclear or misleading conclusions about the effectiveness of the intervention and is considered to contribute to ‘research waste’ [10]. The true test of the effectiveness of an intervention requires a high level of intervention fidelity.

School-based prevention programmes are complex interventions, with multiple components designed to work synergistically, therefore process evaluations which analyse implementation can aid the interpretation of complex outcome effects and understanding of intervention theory [11, 12]. Programme integrity in school-based interventions is improved when there is recognition by school heads, administrators, teachers and support staff of the programme’s utility and practicability within an already full curriculum [13, 14]. Not only is it necessary that the school’s staff ‘buy in’ to the programme, but it is equally important that delivery personnel are adequately supported to deliver the programme as designed in terms of, resources and training. School-based interventions often compete for class time and with the demands of the UK National Curriculum, which may lead to incomplete and/or poor delivery [14, 15].

Analysis of the extent to which interventions are implemented with fidelity requires the assessment of a number of elements. These include i) whether intervention components are delivered as prescribed (adherence to form), ii) the amount of exposure to intervention content (dose/uptake), iii) quality of intervention delivery (spirit), iv) participant responsiveness (engagement) and v) programme differentiation (are there some intervention components which are more essential than others?) [7]. Assessment therefore, requires a mixed methods approach, using both quantitative and qualitative methods in order to understand processes which influence implementation, and their variation across contexts [16]. Assessing all these elements enables researchers to understand both the differential patterns of uptake and engagement overall as well as those patterns associated with participant characteristics such as gender, socio-economic status as well as by personnel and sites. This enables more accurate inferences to be made about programme effectiveness, and (if appropriate) any implications for wider roll out/ implementation [7].

The Healthy Lifestyles Programme (HeLP) trial was a definitive cluster randomised controlled trial involving state primary schools in the South West of England. HeLP was designed to prevent obesity by changing patterns of child behaviour through the creation of supportive school and home environments. The programme consisted of a dynamic, evolving set of processes, using highly interactive delivery methods that encourage identification with, and ownership of, healthy lifestyle messages related to unhealthy snacking (including drinks) and being more active. The intervention took a holistic settings-based approach (in line with the World Health Organisation’s Health Promoting Schools framework) with a focus on the curriculum, the school ethos/environment and links with families/communities [17]. The building of relationships with teacher, pupils and families was central to the programme and careful consideration was given regarding the spirit in which HeLP should be delivered such that it supports the building of these relationships and engages participants across the socio-economic spectrum. The HeLP intervention used the Information Motivation and Behavioural skills model [18] as a guide to selecting informational, cognitive and skill changes that could support behaviour change. Intervention activities were then ordered to enable, support and sustain behaviour change in accordance with the Health Action Process Model (HAPA) [19]. The aims were to motivate children to discuss the key messages relating to snacking and physical activity at home and affect change within the school and family environment [20, 21]. The results of the exploratory trial of HeLP with four primary schools provided proof of concept for these aims [22, 23].

Table 1 shows the intervention phases, components, the theory based behaviour change techniques [24] employed and the delivery personnel. Four HeLP Coordinators were each allocated eight schools (four intervention and four control) and were responsible for overseeing the collection of measurements in both intervention and control schools and the delivery of HeLP in their four intervention schools. HeLP Coordinators were responsible for delivering many components of the programme and were seen as central to building relationships with schools, children and families and supporting teachers throughout the study. They were provided with a written delivery manual and practiced delivery of school assemblies, parent assemblies and goal setting, with critical feedback from the Trial Manager and the parent representatives on the Project Advisory Group (PAG), prior to delivering the components to the children. A Drama Facilitator led the drama sessions and coordinated delivery of the activities for each drama session in the Healthy Lifestyles Week (phase 2). No training was required for the teachers to deliver the lessons and each lesson plan was set out clearly with objectives linking to the National Curriculum at Key Stage 2. Teachers were provided with a teaching manual containing all five lesson plans and associated resources in both hard and electronic form. Actors and Drama Facilitators were University or College graduates in drama or applied drama and ranged in age between 23 and 26 years. All actors and drama facilitators completed a four-day training programme and were given a detailed manual of the drama scripts and an overview (verbal presentation and written document) of the HeLP intervention and all were paid for their training and delivery. The Drama Facilitator for each drama team (comprising four actors) coordinated practise sessions prior to delivery in each school. The HeLP Coordinator worked closely with each drama group in their designated intervention schools to ensure that delivery was well coordinated and that they received constructive feedback after each session to enhance quality and fidelity of delivery. The HeLP Coordinators also received advice/support and feedback regarding delivery of the programme from the Trial Manager throughout the duration of the study. This allowed for an open forum for both the actors and the HeLP Coordinators to discuss their delivery experiences and allowed for suitable strategies to be put into operation to overcome any obstacles.

**Table 1:** Behaviour change techniques (BCTs), components and delivery personnel by intervention phase

Phase	Behaviour Change techniques (BCTs)	Component (Frequency and Duration)	Delivery personnel
**Phase 1** **Creating a supportive context** **Spring term of school Year 5 (9-10 year olds)** **Jan-March**	• Provide information on behaviour-health link • Provide information on health behaviour link • Modelling/demonstrating behaviour • Prompt identification as a role model • Provide information on behaviour-health link • Skill building	Whole school assembly (1x20 mins) Newsletter article Literacy lesson (to create HeLP rap) (1x1 hour) Activity workshops (2x1.5 hours)^b^ Parent assembly (1x1 hour) involving child performances^a^	HeLP Coordinators HeLP Coordinators Class teacher Professional sportsmen/dancers Class teachers/HeLP Coordinator/Drama group
**Phase 2** **Intensive Healthy Lifestyles Week – one week** **Summer term of school Year 5 (9-10 year olds)** **April-June**	• Provide information on health behaviour link • Problem solving/barrier identification • Modelling/demonstrating behaviour • Prompt identification as a role model • Communication skills training • Teach to use prompts/cues	Education lessons (5x1 hour) (morning) Drama (5x2 hours) (afternoon)^b^ (forum theatre; role play; food tasting, discussions, games, etc).	Class teacher Drama group
**Phase 3** **Personal Goal Setting with Parental Support** **Summer term of school Year 5 (9-10 year olds)** **June-July**	• Self-monitoring • Goal setting (behaviour) • Problem solving/barrier identification • Plan social support • Provide information on where and when to perform a behaviour • Agree behavioural contract • Prompt identification as a role model	Self-reflection questionnaire (1x40 mins) Goal setting sheet to go home to parents to complete with child (1x10 mins)^c^ 1:1 goal setting interview (1x10 mins) (goals sent home to parents) Forum theatre assembly (1x1 hour)^a^	HeLP Coordinator HeLP Coordinator/Parents HeLP Coordinator HeLP Coordinator/Drama group
**Phase 4** **Reinforcement Activities** **Autumn term of school Year 6 (10-11 year olds)** **Sept-Dec**	• Provide information on health behaviour link • Modelling/demonstrating behaviour • Prompt identification as a role model • Provide social approval • Prompt self-monitoring • Prompt intention formation • Follow up prompts • Prompt review of behavioural goals • Prompt barrier identification and resolution • Coping plans	Education lesson (1x1 hour) Drama workshop (1x1 hour). Followed by a class delivered assembly about the project to rest of school (1x20 mins) 1-to-1 goal supporting interview to discuss facilitators/barriers and to plan new coping strategies (1x10 mins) (renewed goals sent home to parents)	Class teacher HeLP Coordinator/Drama group HeLP Coordinator

^a^ Formal parental engagement event

^b^ Invitation for parents to observe

^c^ Required parental involvement to complete task

Although HeLP consisted of specified components, designed to be delivered in a prescribed order (see Table 1), the programme was also designed to allow for some degree of flexibility and adaptability to the school context. The HeLP Coordinator worked closely with each school throughout the year-long intervention to understand how best to engage and involve parents and children, as well as how best to deliver components within the constraints of the timetable and available space.

**Fig. 1:**
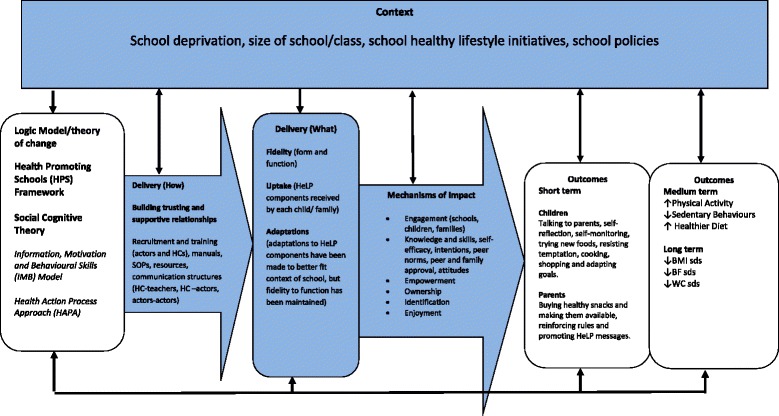
Key functions of the process evaluation for HeLP

The wider process evaluation of the definitive cluster randomised controlled trial of HeLP was informed by the Medical Research Council’s guidance on process evaluations of complex interventions [16]. This paper reports on the quantitative and qualitative data collected to assess the implementation of the HeLP intervention in the sixteen intervention schools (see Fig. 1).

## Methods

The HeLP study was a pragmatic, superiority, school-based, cluster randomised controlled trial with blinded outcome assessment, conducted in Devon in the South West of England. It ran from March 2012 to November 2016 [25] and, for practical reasons, was delivered in two cohorts (Cohort 1 commenced the trial in September 2012 and cohort 2 in September 2013). The intervention was delivered from January 2013 to November 2014 in cohort 1 and January 2014 to November 2015 in cohort 2. Thirty two schools (with 1324 Year 5 children recruited) which represented a range of school sizes (one to three year 5 classes), locations (urban and rural) and deprivation (<19% and ≥19% of children eligible for free school meals) took part. Sixteen schools were randomly allocated to the intervention group and 16 to the control group. The published trial protocol [25] and statistical analysis plan [26] give further details.

### Ethics and consent

Ethical approval for the trial was obtained from the Peninsula College of Medicine and Dentistry in March 2012 (reference number 12/03/140). Children were recruited using an ‘opt out’ system in which detailed written information about the trial was sent directly home to parents/carers via the school, with parents returning an ‘opt out’ form if they did not wish their child to participate either in the measures only or both the intervention and the measures. Parents were given three weeks in which to return the opt-out form and class teachers made regular reminders to the children during this period to encourage their parent(s) to read the pack. Parents were able to speak to the class teacher or the school’s allocated HeLP Coordinator at any time if they required further information, which was also made clear in the provided written information [25]. All children who were on the registration list at one of the recruited schools at the start of the Autumn term 2012/13 and whose parent/carer did not complete an opt-out form, were classed as participants.

### Intervention fidelity

Intervention fidelity was assessed under the following headings: intervention uptake (exposure/dose), adherence to intervention components (fidelity to form), quality of intervention delivery (fidelity to the spirit of the intervention) and participant responsiveness to the intervention as a whole (engagement of the whole school, teachers and children). We employed a number of methods to promote intervention fidelity, these included continuing involvement from our stakeholder group regarding intervention delivery, consideration of delivery qualities and characteristics of personnel in recruitment of delivery personnel, training and manualising delivery for every component based on extensive piloting. We did not assess programme differentiation (whether some components are more essential than others), as complex interventions which have multiple components are designed to work synergistically [27]. HeLP was dynamic and evolved a set of processes with reinforcing feedback loops between the school, child and family, so assessing whether some intervention components were more essential than others would fail to account for the inherent synergy between components and across phases [28].

#### Intervention uptake

Registers of attendance were kept by the HeLP Coordinator for all intervention components and for parental attendance at parental engagement events (i.e. parents invited to the school to observe activities). The Year 5 teachers were asked to keep a register for teacher delivered components when the HeLP Coordinator was not present (these included the literacy rap lesson in phase 1 and the education lessons in phases 2 and 4). The completed checklist was then given to the HeLP Coordinator.

#### Adherence to intervention components

Adherence was assessed using yes/no checklists to indicate whether subcomponents within each component had been delivered or not (see Additional file 1 for an example checklist). These were completed by the HeLP Coordinator or the Year 5 class teacher (for teacher delivered components only). In order to minimise the potential for bias, the class teacher was given a content checklist to observe and score one component within their school which was completed independently of the HeLP Coordinator. These were then compared for discrepancies.

Teacher completed checklists were returned to the HeLP Coordinator. If all components for each phase were delivered as per the manual (represented as a tick on the checklist) then it was recorded that 100% of HeLP components had been delivered in that school. If a minor activity (e.g. the practise of a chant, a scene from a drama workshop) was missing from a particular component, 2% was deducted for each minor activity from the overall total. See Table 2 for further details of data collection and analysis.

**Table 2:** Details of fidelity data collected and analysed in the HeLP cluster RCT

Element of fidelity assessed	Method of data collection	Data collected	Number completed	Time of data collection	Personnel collecting data
**Intervention uptake (dose/exposure)**	Registers	Child attendance for all intervention components and parental attendance for formal parental engagement events and invitations to observe (see Table 1).	21/21 child attendance registers completed for each of the 16 intervention schools for children. 4/4 parental attendance registers completed for each of the 16 intervention schools.	During delivery of each intervention component.	HeLP Coordinator and Teachers for the literacy lesson in phase 1 and education lessons in phase 2 and 4.
**Adherence to intervention components (form)**	Observations and completion of checklists	Each component for each phase of the intervention had a number of sub components. A yes or no was given depending upon whether the subcomponent had been delivered.	100% (71/71 subcomponents assessed in each of the 16 intervention schools)	During delivery of each intervention component.	HeLP Coordinator Year 5 teachers (for those components delivered solely by the Year 5 teacher): • Literacy rap lesson in phase 1 • Education lessons in phase 2 and 4
**Quality of intervention delivery (spirit)**	Observations and completion of checklists	Quality of delivery and child, parent and teacher response scores (1–10) during delivery of the following components: • Parent assembly (phase 1), • Healthy Lifestyles Week (phase 2), • Parent assembly (phase 3) • Class delivered assembly (phase 4).	100% (4/4 components assessed in each of the 16 intervention schools) 128 observations in total	During delivery of the specified intervention component.	Trial Manager (phases 1, 3 and 4) HeLP Coordinator (phase 2)
**Participant responsiveness to the intervention as a whole (engagement)**					
**Quantitative assessment**	Observations and completion of field notes	Engagement scores: School (range 0–9) Child (range 0–3)	96% (16/16 schools652/676 children assessed)	Finalisation of scores were made immediately post intervention (December 2013/14 for Cohort 1/2).	HeLP Coordinator
**Qualitative assessment**	Teacher interviews and child focus groups	Teacher and child experience of participating in HeLP	28 teacher interviews and 35 child focus groups	June/July 2013/14 for Year 5 teacher interviews (following phase 3 of the intervention prior to schools breaking up for the Summer holiday)	Trial Manager
				Jan-March 2014/15 for child focus groups (when the children were in year 6)	Led by the HeLP Coordinator for that school and facilitated by an additional HeLP Coordinator, who took notes and supported the management of the group.

#### Quality of intervention delivery

Four components were chosen to assess quality of intervention delivery (one in each phase). These were the parent assembly (phase 1), the Healthy Lifestyles Week (phase 2), the parent assembly (phase 3) and the class delivered assembly (phase 4). For each of the four components, a score between 1 and 10 was given for (i) delivery; (ii) child response; (iii) parent response and (iv) teacher response. The criteria used to assess the quality of delivery were enthusiasm, open body language, responsivity to child/school needs and clear and friendly communication. The criteria to assess participant response to the delivery were attentiveness, positive body language (e.g. smiling, open posture) and active involvement (when required). These criteria evolved from the extensive piloting of the programme and in discussion with the advisory group and were felt to encompass the ‘spirit’ of the Healthy Lifestyles Programme.

The Trial Manager and the Principal Investigator independently scored quality of intervention delivery for the parent assembly in phase 1, across three schools. No discrepancy in scoring was observed for either quality of delivery or participant response. Thereafter, the majority of the observations were carried out by the Trial Manager.

The HeLP Coordinators assessed all the five drama sessions in the Healthy Lifestyle Week components (phase 2), after having carried out initial assessments alongside the Trial Manager (see Additional file 2 for an example checklist). Again, in order to control for possible bias, the Director of Headbanger’s Theatre Company, who had developed the drama scripts with the Trial Manager, observed and scored quality of delivery for one drama session in each of the 16 schools independently of the HeLP Coordinator. Scores were then compared for discrepancies.

The four scores (quality of delivery and child, parent and teacher response) per observation in each school were averaged (mean score) to create a single summary score out of 10 for each of the four components observed. These four component scores were then averaged to produce a single mean delivery score per school. A score ≥ 8 was pre-specified to indicate that the intervention had been delivered in the ‘spirit’ in which it had been designed.

#### Participant responsiveness to the intervention overall

Levels of engagement with the programme overall were assessed both quantitatively and qualitatively for schools and for individual Year 5 teachers and children in intervention schools.

##### Quantitative assessments

Assessment of a child’s engagement was based on field notes collected during the interaction of the HeLP Coordinator with the child during the 1–1 goal setting interview and from more general observations during the course of the intervention. The HeLP Coordinator gave each child an engagement score between 0 and 3 (see Table 3 for the criteria for scoring). Individual scores were dichotomised to create two groups (≤ 1 indicating less engaged children and >1 indicating engaged children).

**Table 3:** Scoring criteria for child and school engagement

**Scoring criteria for child engagement**
0 = disinterested/unaware goals needed to be set
1 = reluctant/needs a lot of prompting
2 = enthusiastic and happy to chat about goals and how they will achieve them
3 = very enthusiastic, has discussed them at home and has clear strategies for achieving them
**Scoring criteria for school engagement**
0 = unengaged/uncooperative
1 = supportive
2 = enthusiastic and supportive
3 = very enthusiastic and used HeLP in other aspects of teaching/school activities

Child level engagement was analysed by gender and by socio-economic status using quartiles of the child’s Index of Multiple Deprivation (IMD) score, as determined by the child’s home postcode [29].

School level engagement was assessed based on the HeLP Coordinator’s interaction with, and observations of, the head teacher, the Year 5 teacher(s) and the school support staff. A score between 0 and 3 was given to each staff member, (see Table 3 for the scoring criteria). Individual scores for the head teacher, Year 5 teacher and the support staff were aggregated to give a score out of 9 for each school. Schools were then dichotomised into two groups (0–3 = less engaged school and 4–9 = engaged school). As a school could score 3 if the Year 5 teacher, headteacher and support staff were all considered ‘supportive’ we a priori agreed that in order to be categorised as engaged at least one member of staff needed to demonstrate enthusiasm for the Programme.

##### Qualitative assessments

All Year 5 teachers from each of the 16 intervention schools participated in a 40–45 min semi-structured interview on the school premises. All children were eligible to be selected for the focus groups. The HeLP Coordinator selected children to participate in a 60 min focus group during school time based on the child’s level of engagement (i.e. those considered to be most engaged vs those least engaged with the programme). Two or three focus groups, each of between 6 and 8 children, were carried out per school depending upon school size. At least one group per school was with children who were categorised as being engaged and one group was with children who were less engaged (with similar numbers of boys and girls, where possible). For each category (engaged vs less engaged), children were selected purposely in order to bring together a group with a mix of boys and girls, weight and socio-economic status. Children and parents were sent a letter informing them that their child had been selected to participate in an interview and parents completed a consent form to allow their child to participate during school time. No incentives were given. In order to help children remember the details of the programme visual cues were provided with a short summary of the activities (see Additional file 3 for teacher interview schedule and Additional file 4 for child focus group schedule).

Interviews and focus groups were digitally recorded and verbatim transcripts prepared from the sound files. The transcripts were checked for accuracy against the sound files and corrections were made if required. Any comments that could identify people or schools were anonymised before the transcripts were imported into NVivo. Thematic content analysis [30] identified themes which were developed into an analytic framework, based on the process evaluation aims, interview guides and additional themes identified during the analysis process.

Four transcripts from the cohort 1 focus groups were selected (two engaged and two less engaged groups) from each HeLP Coordinator’s group of schools and read by the cohort 1 HeLP Coordinators and Trial Manager and an initial code framework was agreed. Minor edits were made to the topic guide for the cohort 2 focus groups. A similar process was used with the cohort 2 focus groups and the initial codes were discussed, refined or amended and a new coding framework produced by HeLP Coordinators and Trial Manager. This coding framework was then used by the HeLP Coordinators and one independent researcher to code the remaining focus groups. The teacher interviews were coded using a similar approach with the coding framework being edited at each stage. The Trial Manager coded 20% of these transcripts (*n* = 13), with the Principal Investigator providing verification for half of the 20% coded by the Trial Manager. The codes were then categorised (second cycle coding) to identify emerging themes and sub-themes. Data from teachers and children were collated for each theme/sub-theme and transferred into tables. The resulting tables were then analysed to look for agreement, partial agreement, silence or disagreement between participants and the different data sources.

All quantitative data were entered into a Microsoft Access® (2014 version, Microsoft Corporation, Redmond, WA, USA) database. The data were then exported into Microsoft Excel® (2014 version, Microsoft Corporation, Redmond, WA, USA). All qualitative data were entered into NVivo (Version 11, QRS International, Warrington, UK).

Table 2 provides further details of data collection and analysis for each element of fidelity assessed.

## Results

Thirty two schools participated in the trial with a total of 1324 children (676 children in the intervention group and 648 in the control group). The HeLP intervention was delivered to children in 16 schools (7 of which had ≥19% of children eligible for free school meals) and 27 classes in total.

### Intervention uptake

Over 90% of children participated in each phase of HeLP and 93% of children received the four drama sessions in phase 2 and the 1–1 goal setting in phase 3 (the components focusing intensively on individual level behaviour change) delivered in the ‘spirit’ of HeLP. (see Table 4).

**Table 4:** Uptake of HeLP

	phase 1	phase 2	phase 3	phase 4	Percentage of children receiving 4 drama sessions (phase 2) and the goal setting (phase 3)^a^ delivered in the spirit of HeLP^b^
Cohort 1	91.2%	94.1%	91.1%	92.1%	93.7%
Cohort 2	94.7%	93.7%	92.5%	91.4%	92.7%
Total	93.4%	93.9%	92.0%	91.6%	93.0%

^a^ Components focusing intensively on individual behaviour change

^b^ *Enthusiastic delivery, open body language, responsive to child/school needs and clear and friendly communication*

### Adherence to intervention components and quality of delivery

No discrepancies were seen between the HeLP Coordinator’s assessment of fidelity to intervention content and those of the independent assessor. Equally no discrepancies were observed between assessors for fidelity to quality of delivery for either the four components selected for assessment. Table 5 shows that we can be confident that all intervention schools received a complete or near complete programme which was delivered as designed (i.e. all schools scored over 8 for quality of delivery for the 4 components observed).

**Table 5:** Adherence to intervention components and quality of delivery

School Cohort 1 (1–8)Cohort 2 (9–16)	% of components delivered in complete form	Quality of delivery score (Max score 10)
1	100%	8.9
2	100%	9.0
3	100%	9.1
4	100%	9.8
5	96%	9.7
6	94%	9.4
7	96%	9.5
8	98%	9.6
9	94%	9.0
10	94%	9.1
11	96%	9.4
12	96%	9.7
13	98%	9.2
14	96%	8.1
15	100%	9.7
16	94%	9.2

### Participant responsiveness to the programme overall

School and child engagement scores as well as the qualitative data from the focus groups and teacher interviews relating to enjoyment and engagement of the programme are presented together. Evidence from teachers and children relating to programme engagement/enjoyment (e.g. knowledge and understanding, comments on content/delivery methods, including identification with the characters) are also presented. Quotes are presented in boxes and referenced with the source: School number, T = teacher, LEC = less engaged child, EC = engaged child. No child was opted out of the focus group by their parents.

#### Child engagement

Ninety six percent (652/676) of children set goals with the HeLP Coordinator in phase 3 and of these children, 63% (411/652) had parental support, as indicated by a parent signature on the goal setting sheet and/or written comments regarding how the parent would support the child in achieving their goals. Twenty four children (4%) had missing engagement scores: 13 children had moved out of the area; 8 children had changed schools prior to the 1–1 goal setting discussion; and 3 children were absent from school on multiple visits by the HeLP Coordinator and therefore had not set goals.

Based on the HeLP Coordinator’s assessment, 92% (602/652) of children were deemed to be engaged with HeLP. Similar percentages of boys and girls were considered engaged (91% and 94% respectively); however, there was greater child-level engagement in schools with more than one Year 5 class compared to schools with only one Year 5 class (97% and 82% respectively). Table 6 shows that HeLP was able to engage children across the socio-economic spectrum, although in the less engaged category, there was a higher proportion of children from the most deprived quartile compared to the least deprived quartile.

**Table 6:** Child engagement by Index of Multiple Deprivation rank^a^

Deprivation Quartile	Number (and %) of less engaged children (LEC)	Number (and %) of engaged children (EC)	Total number of children
1 (most deprived)	16 (33)	156 (26)	172
2	15 (31)	143 (24)	158
3	8 (16)	147 (24)	155
4 (least deprived)	10 (20)	155 (26)	165
Total	49	601	650

^a^Two children could not be included in the analysis of engagement by IMD rank as we did not have their postcodes

All Year 5 teachers from the 16 intervention schools were interviewed (*n* = 28) and a total of 35 focus groups (18 with engaged children and 17 with less engaged children) of 6–8 children in each group were conducted across both cohorts.There was very clear evidence from the teacher interviews and the child focus groups that children really enjoyed and engaged with all aspects of the the programme across all schools. All children spoke positively about HeLP:

*‘I thought it was much more different than I thought it would be, because I didn’t really exactly imagine it as a boring old literacy lesson or anything but I didn’t really think it was going to be that joyful and exciting. It was much more than I expected.’* **(female LEC, school 14)**

*‘Amazing, fun, healthy, extraordinary and the best!’* **(female EC, school 7)**

*‘It was brilliant it was such good fun; the children reacted to it really positively. In fact I have not heard them say anything negative about it at all. They’ve never said ‘oh no we’re doing this again’ they’ve always been really open and engaged….’* **(T, school 15)**

The Healthy Lifestyles Week seemed to be the most enjoyable aspect, with the majority of children reporting that they liked the interactive and dynamic nature of the sessions and working with the actors and characters, with whom they identified.

*'we had lots and lots of fun and everyone was getting excited for the next day and the next and the next, and for that week of activities, I was really excited about actually going to school, usually I’m just ‘oh I want to stay in bed’ but that week I was really excited about going to school.‘* (**male LEC, school 12**)

*‘the reason I liked the Healthy Lifestyles Week was because you were actually seeing what, sort of like a made up version of four different people who have trouble and the ways you can improve it by just following them.’* **(female LEC, school 12)**

*‘um, my favourite part ……. was probably in the drama was like when we were, because it was so totally different to what we normally do. In school we obviously do literacy and maths when we have to write stuff down but you could really express your emotions through drama, I really liked that.’* **(female EC, school 6)**

However there were a minority of children who were less keen on the acting aspect:

*‘I didn’t like doing the acting but it was ok when we were doing the non-acting.’* (**male EC, school 3)**

*‘well they were really good but when the characters asked us to come up do a bit of acting and stuff it was a bit embarrassing, because I put my hand up and I didn’t know what I was in for.’* (**female LEC, school 9**)

The teachers reported that the delivery of the Healthy Lifestyles Week and the activity workshops engaged the children, and in some cases even the children who were usually shy or disruptive.

*‘I thought that the cleverness of the drama was that they did it from a child’s perspective. They acted out as children, but they were like the cool teenage children which children can relate to. Interestingly enough, there are several children in my class that could switch off so easily but they didn’t, because they immediately were drawn in to this character, this role play character. I thought the idea of having the Duncan and the Active Amy and all the rest of it, initially when you heard it you thought oh this isn’t going to work, and then you saw them go straight in to role, the acting was fantastic…..’* **(T, school 13)**

*‘Yes, yeah there was one boy who would, well no two of them in fact that would never ever ever get up and do any drama or anything and they were up taking part in everything. I was so touched with emotion I had to run out and tell mum at the end of the day. So I think that it bought him out cos he, he was one of the ones that did it in the hall as well.’* **(T, school 10)**

*‘And the acting just lifted it 100 times more and to them it was so important and to them …we did the lesson in the morning but they couldn’t wait until the afternoon. They were so linked to those characters and it was such a clever thing.’* **(T, school 15)**

Children’s knowledge and understanding of the key messages were considered to provide further evidence that children had engaged with the programme; there was also evidence from the focus groups of their understanding of the messages around marketing, moderation and food labelling.

*‘what helped me a massive amount was looking at the ingredients and looking at what’s inside stuff, like if it says like fruit on it, it may not actually be made out of fruit. Like fruit winders and stuff and some stuff and my mum said like if they make the big front of the packet really appetising and want to make you feel like you want to buy them but then the back is like all small and you can hardly read it so they are trying to trick you to get the really unhealthy stuff but make it look really appetising.’* **(male EC, school 16)**

*‘I think my favourite part about it was doing the food machines. I especially liked how they used acting to show how the foods were made and what process they go through.’* **(male LEC, school 12)**

### School engagement

School engagement scores ranged from 9 (maximum score) to 2. Out of the 16 schools, only three were categorised as ‘less engaged’ according to the school engagement scoring system. Reasons for this included administrative and teaching challenges due a school being placed on special measures by OFSTED during delivery and staffing issues due to the absence of the head and/or teachers, whereby the workshops were viewed as an opportunity to free up teachers to do ‘other things’.

Table 7 provides a summary of each school by cohort in relation to size, deprivation, location and engagement. The UK Government guidelines [31] define populations less than 10,000 as rural. For the purposes of this study we defined schools located in small towns as urban/rural. There were no differences in engagement by location, % pupils eligible for free school meals or number of Year 5 classes.

**Table 7:** School engagement information

*School Cohort 1 (1-8) Cohort 2 (9-16)*	*Number of Year 5 classes*	*% FSM (National average 19%)*	*Urban/rural*	*School staff engagement score*	*Overall school engagement score*
1	1	<19%	Urban/rural	Head = 2, Teacher = 0, Admin = 1	3
2	2	>19%	Urban	Head = 1, Teacher = 1, Admin = 2	4
3	1	>19%	Urban	Head = 0, Teacher = 1, Admin = 1	2
4	2	>19%	Urban	Head = 1, Teacher = 3, Admin = 2	6
5	1	>19%	Urban/rural	Head = 1, Teacher = 2, Admin = 3	6
6	1	>19%	Urban	Head = 1, Teacher = 2, Admin = 2	5
7	1	>19%	Urban	Head = 3, Teacher = 3, Admin = 1	7
8	1	<19%	Rural	Head = 2, Teacher = 2, Admin = 1	5
9	2	<19%	Urban	Head = 2, Teacher = 3, Admin = 3	8
10	3	<19%	Urban	Head = 1, Teacher = 2, Admin = 2	5
11	3	<19%	Urban/rural	Head = 1, Teacher = 3, Admin = 2	6
12	2	<19%	Rural	Head = 2, Teacher = 2, Admin = 2	6
13	1	<19%	Urban	Head = 3, Teacher = 3, Admin = 2	8
14	2	<19%	Urban	Head = 0, Teacher = 1, Admin = 2	3
15	1	>19%	Rural	Head = 3, Teacher = 3, Admin = 3	9
16	3	<19%	Urban	Head = 2, Teacher = 2, Admin = 2	6

## Teacher engagement

Regardless of the HeLP Coordinator’s perception of teacher engagement (see Table 7) all Year 5 teachers spoke positively about the programme during their interviews and there was strong evidence that teachers were engaged. Many believed that the programme’s compatibility with the National Curriculum made it feasible to deliver and helped reinforce healthy lifestyle messages that the curriculum already promotes. All teachers reported that the HeLP Coordinator assigned to their school provided excellent ongoing support, which really helped in the delivery of the programme. Many teachers commented that the way in which the lessons were delivered and the personnel used for delivery were central in helping the children think more deeply about their health and how they could make small achievable changes to their eating and activity behaviours.

*She’s [HeLP Coordinator] ever so good, she’s obviously worked with teachers quite a lot I think, she knows how it is. I don’t have to say ‘oh because we’re doing this’ she says ‘oh yeah yeah I realise so you know she’s really really good at fitting the scheme, we’re trying to fit in with you and she helped fit in with us so you know it worked really well. That flexibility is really important and you know she’s brilliant and she knows her stuff and she knows… I’ve been really impressed she’s been brilliant, really helpful and ever so amenable. We’ve had children with possible problems with their eating and you know she was brilliant talking to the parents……… nothing was too much trouble it was like ‘oh yeah I’ll sit down and chat with them’ and she was brilliant when she was sort of talking to the parents and I think it is really important that you have that point of contact. For me it was so easy that I could you know send an email or [HeLP Coordinator] would email me and I could email back and you know that’s really really useful and very good and helps the thing run really well.’* **(T, school 15)**

*‘ I think they really engaged with those lessons. You know I was thinking about some of the messages that were coming across from the actors, when we had the actors come in for that week you know we tell them those messages all the time and they don’t sink in as much as they did then. And I think that coming from a younger perspective, the street dance obviously and [name of sports group] you know they nailed it really. Yeah they loved that and yeah I think you know its one thing having, I’ve got great relationships with the kids we all have but its one thing having Mrs xxxxx tell them this and its quite another thing to experience it from younger people, or people they perceive to be younger. The actors were teenagers and they loved them. So yeah I think it had a deeper impact because it came from a different perspective. Than their parents or their teachers really.*’ **(T, school 11)**

## Discussion

Scaling up from an exploratory trial poses many challenges to researchers in terms of intervention fidelity in the definitive effectiveness trial [9]. We had shown the feasibility and acceptability of the intervention and the trial processes in an exploratory trial involving four schools, two of which received the intervention [22]. However, the definitive trial involved 32 schools and an eight-fold increase in intervention delivery. Continuing involvement from our stakeholder group regarding intervention delivery [32], consideration of delivery qualities and characteristics of personnel [20], training and manualising delivery for every component based on extensive piloting [22, 33] supported the high degree of intervention fidelity across all schools and by all four HeLP Coordinators.

Over 90% of children attended all intervention components in each phase (uptake) and received 94–100% of programme of activities (adherence to intervention components) delivered in the spirit in which HeLP had been designed (quality of delivery). In addition, both quantitative and qualitative assessments of child, school and teacher engagement revealed that there was a high degree of participant responsiveness to the intervention as a whole at both the school and child level. Qualitative data showed that children responded very well to the intervention, enjoying the dynamic and participatory nature of the delivery methods and teachers thought the intervention was relevant, acceptable and well delivered by the external personnel.

Quantitative data showed that 93% of children were engaged with no differential engagement across the socio-economic spectrum. This is a very positive finding as there is concern that engagement with school-based interventions to improve children’s health and subsequently their effects may favour the healthiest, potentially perpetuating existing health disparities [34, 35]. This has been expressed in the literature as the ‘inequity paradox’ [36] or the ‘inverse care law’ [36].

There was no differentiation in child engagement by gender, however, a small difference was observed by school size (1 class vs more than one class), with more children deemed to be engaged in the larger schools. Although we recognise that the engagement measure was somewhat subjective, this difference could be attributed to the ability to create more of a whole school awareness about the programme in schools with more than one Year 5 class. We had hypothesised that school size could be an important covariate/confounder and so school size was one of the stratification variables in the randomisation. Only three schools were categorised as ‘less engaged’ (i.e. scored 3 or less out of a maximum score of 9). Process evaluation data revealed that the reasons for this were based on internal staffing issues rather than aspects of the intervention or its delivery.

Our uptake and adherence to intervention components results compare favourably to other school-based obesity prevention programmes. In the UK Active for Life Year 5 trial [37], although 95% of children in intervention schools received the lessons, a mean of 12.3 lessons were delivered, equating to 77% of the intervention. Out of the 10 homework assignments a mean of 6.2 were set for the children to complete. Similarly, only 64% of components were delivered across all intervention domains in the HEROES obesity prevention programme [38].

We attribute our high intervention fidelity to a number of reasons. The programme was developed over several stages of piloting [33] involving extensive stakeholder engagement in both the design of the intervention and the trial to ensure that it was feasible and acceptable to schools, children and their families. The intervention was designed to meet National Curriculum objectives in Key Stage 2, so that it did not displace the teaching of nationally required learning targets and the use of outside personnel meant that teachers were not over burdened with excessive training, preparation and delivery requirements. This supports findings from the Active for Life Year 5 trial [39] which cites a lack of time and pressure to focus on core literacy and numeracy skills as the main reason for teachers omitting key intervention components. A realist systematic review on implementing health promotion programmes in schools revealed that providing relevant ‘on the ground support’ and training was essential to deliverers, whether they are teachers or other professionals working within a school. For example teachers may need skills and confidence in specific behaviour change techniques that are part of the programme, whereas outside professionals may need skills and confidence in classroom management [15]. The HeLP Coordinators provided support to teachers in coordinating delivery of programme components and all delivery personnel received training, supervision and detailed manuals, including scripts for the delivery of the drama sessions. Indeed, previous research shows that training manuals, with clear descriptions of the activities, are key to the promotion of programme integrity [40, 41], as well as to its training and supervision of delivery personnel [42].

Detailed manuals for delivery and appropriate selection and training of the key delivery personnel (HeLP coordinators and the actors) meant that HeLP was delivered in the spirit in which it had been designed and was able to engage and motivate the children to take the messages home to their parents and make small achievable changes to their eating and activity behaviours. The building of relationships was central to every aspect of the HeLP intervention and our engagement data reflect a high level of buy in from schools staff. Studies examining factors associated with health promotion programmes in schools found that teachers’ attitudes concerning the intervention itself (i.e. whether they liked it and/or thought it was worthwhile or not) and the training and support they themselves received, were all associated with their level of adherence to the intervention [43, 44].

### Study strengths and limitations

As part of the HeLP trial, we undertook a comprehensive assessment of intervention fidelity using multiple sources of data, as recommended in the MRC guidance on process evaluation of complex interventions [45]. We developed detailed manuals and checklists and triangulated both qualitative and quantitative data from multiple sources to assess engagement with the programme, in addition to ‘uptake’ (i.e. the dose received/reach), providing a more nuanced and comprehensive picture of intervention fidelity. Although we used pre-specified criteria to assess child and staff engagement, we acknowledge that a limitation is the subjective nature of these data.

As it was not feasible or practical to assess the quality of delivery for all intervention components, we selected four which enabled us to assess both child and teacher response. These observations were carried out by trained researchers who had been involved in intervention development and who had carefully considered the qualities associated with the spirit of intervention delivery. Adherence to intervention components was assessed by an independent observer, increasing the validity of the data; however, we relied on self-assessment of content delivery for the six teacher delivered lessons, which is less robust.

## Conclusion

The use of a pragmatic trial design (which aimed to assess delivery under ‘real world’ settings) and analysis of the factors influencing fidelity to delivery provide valuable evidence to aid interpretation of trial findings. Our trial shows that it is possible to successfully scale up complex school-based interventions, engage schools and children across the socio-economic spectrum and deliver an intervention as designed. As programme integrity was maintained throughout the HeLP trial, across all intervention schools, we can be confident that findings will be a true reflection of the effectiveness of the intervention, enabling policy recommendations to be made.

## Additional files

Additional file 1: Fidelity to delivery (content) checklist. (DOCX 13 kb)

Additional file 2: Fidelity to delivery (quality) checklist. (DOCX 16 kb)

Additional file 3: Teacher interview schedule. (DOCX 16 kb)

Additional file 4: Child focus group schedule. (DOCX 16 kb)

## Abbreviations

BMI: Body Mass Index
EC: Engaged child
FSM: Free school meals
HAPA: Health Action Process Model
HeLP: Healthy Lifestyles Programme
IMD: Index of Multiple Deprivation
LEC: Less engaged child
PAG: Project Advisory Group
RCT: Randomised Controlled Trial
WHO: World Health Organisation
