# Supplementary material for: Intervention fidelity in the definitive cluster randomised controlled trial of the Healthy Lifestyles Programme (HeLP) trial: findings from the process evaluation
Source: Int J Behav Nutr Phys Act. 2017 Nov 28;14:163. doi: 10.1186/s12966-017-0616-6 (PMC5704582; doi:10.1186/s12966-017-0616-6)
Supplement: Supplementary file 1 — Fidelity to delivery (content) checklist. (DOCX 13 kb) [file 12966_2017_616_MOESM1_ESM.docx]

**Additional File 1 – Adherence to intervention components checklist.**

**Component - Parents’ assembly in Phase 1**

NAME OF SCHOOL:

NAME OF HC:

DATE:

Adherence to sub-component content

| **Activity – class teacher present** | **Yes/No** | **Notes** |
| --- | --- | --- |
| Intro with rationale of the Programme |  |  |
| Summary of the HeLP Programme |  |  |
| Family invitations |  |  |
| Contacts details given |  |  |
| Dance performance |  |  |
| Sport performance (e.g.rugby/basketball) |  |  |
| Raps performance |  |  |

General comments:
