# Supplementary material for: Intervention fidelity in the definitive cluster randomised controlled trial of the Healthy Lifestyles Programme (HeLP) trial: findings from the process evaluation
Source: Int J Behav Nutr Phys Act. 2017 Nov 28;14:163. doi: 10.1186/s12966-017-0616-6 (PMC5704582; doi:10.1186/s12966-017-0616-6)
Supplement: Supplementary file 2 — Fidelity to delivery (quality) checklist. (DOCX 16 kb) [file 12966_2017_616_MOESM2_ESM.docx]

**Additional File 2 – Quality of intervention delivery and participant checklist**

**Component - Parents’ assembly in Phase 1**

NAME OF SCHOOL:

NAME OF OBSERVER:

DATE:

Quality of delivery and participant response (1=Low 10=High)

|  | **1** | **2** | **3** | **4** | **5** | **6** | **7** | **8** | **9** | **10** |
| --- | --- | --- | --- | --- | --- | --- | --- | --- | --- | --- |
| Enthusiastic delivery |  |  |  |  |  |  |  |  |  |  |
| Engaged/participating children |  |  |  |  |  |  |  |  |  |  |
| Engaged parents |  |  |  |  |  |  |  |  |  |  |
| Engaged school staff |  |  |  |  |  |  |  |  |  |  |

**Deliverer**

- Enthusiastic,
- Open body language,
- Responsive to child/school needs
- Clear and friendly communication.

**Participant response**

- Attentive
- Positive body language (e.g. smiling, open posture)
- Active involvement (when required)
