# Supplementary material for: Intervention fidelity in the definitive cluster randomised controlled trial of the Healthy Lifestyles Programme (HeLP) trial: findings from the process evaluation
Source: Int J Behav Nutr Phys Act. 2017 Nov 28;14:163. doi: 10.1186/s12966-017-0616-6 (PMC5704582; doi:10.1186/s12966-017-0616-6)
Supplement: Supplementary file 3 — Teacher interview schedule. (DOCX 16 kb) [file 12966_2017_616_MOESM3_ESM.docx]

# Additional File 3 - Year 5 Teacher Interview Schedule

**Year 5 Teacher Interview – Intervention Schools**

**INTRODUCTION**

Thank you for agreeing to give a brief interview on your experience of your involvement in HeLP. Understanding your experience of HeLP is vital to the further development of the programme, so please feel free to be as frank as you wish! This interview will be relatively unstructured to allow you to just talk about your views and experiences of participating in the programme. So that I can concentrate on what you are saying is it ok for me to record this interview? All your comments will remain anonymous and will only be used for the purposes of this research project.

1. How did you feel when you heard that your Y5 class was going to receive the Healthy Lifestyles Programme?
   1. Was there anything that made you anxious?
   2. Was there anything that you were excited about?
   3. Is there anything we could have done to allay any fears in the first instance?
2. Do you think it is necessary to have programmes like HeLP given everything else going on in schools?
   1. Probe added value – what makes HeLP distinct
3. Having been through the whole year 5 programme could you give us some indication of the workload/hassle factor?
   1. Has it added to your workload and, if so, in what way?
   2. What could we do differently to alleviate your workload?
   3. Probe how the PSHE lessons were received
   4. How much do the HeLP activities overlap with the year 5 curriculum? (try and get teachers to give a percentage overlap)
4. One of the key roles of the HeLP Coordinator is to build relationships with schools, children and families and especially to support you.
   1. Did you feel supported?
   2. Was there more we could have done?
5. How do you think the children found the programme?
   1. How many drama sessions did you observe and what did you think?
   2. Did you notice any impact at a class/individual child level?
      1. Probe possible reasons for teacher observations re whole class or individual children (need to tease out what is it about HeLP that led to certain behaviours – programme differentiation)
6. Do you think HeLP is known/understood in the wider school context?
   1. Probe examples
7. Do you have any sense of how parents perceived the programme?
8. Have you used any aspect of HeLP in your day to day teaching and/or interactions with the children?
9. Had HeLP had any effect on you personally?
10. Would you recommend HeLP to your colleagues or other schools?
11. Is there anything else you would like to say/comment on?

Thank you so much for sharing your views about the Healthy Lifestyles Programme today.
