# Supplementary material for: Intervention fidelity in the definitive cluster randomised controlled trial of the Healthy Lifestyles Programme (HeLP) trial: findings from the process evaluation
Source: Int J Behav Nutr Phys Act. 2017 Nov 28;14:163. doi: 10.1186/s12966-017-0616-6 (PMC5704582; doi:10.1186/s12966-017-0616-6)
Supplement: Supplementary file 4 — Child focus group schedule. (DOCX 16 kb) [file 12966_2017_616_MOESM4_ESM.docx]

# Additional File 4 - Child Focus Group Schedule

**FOCUS GROUPS– Programme Schools**

Last year you were involved in our Healthy Lifestyles Programme, we are here today to find out what you remember about the Programme and how you felt about it, in research we call this a ‘focus group’ which means to have a chat or discussion about your opinion and feelings about the programme. Each of you is here because we randomly chose names out of a hat. It is really important to get some honest feedback, good and bad, from you as this will help us make the Programme better in the future.

There are no right or wrong answers and we will make sure that each of you has the opportunity to speak- it’s very important we hear everyone’s views. So that we don’t talk over each other we are going to use this carrot, you can talk only when you are holding the carrot when you want to speak put your hand up and we will pass the carrot to you. To help us we are also going to use the ground rules we used during Healthy Lifestyles week, do you remember what they were?

No personal comments

No put downs

Respect and listen to each other

Ok so now we are going to transport you back in time, like the actors did during Healthy Lifestyles week with their time machine, we have some photos here to help remind you of the things we did during the programme and we would like you to look at these and spend some time remembering the activities, thinking about the character you worked with and the goals you set. We will ask questions about your home and your parents and what they thought of the programme too. *Refer to poster-* these are the things we want to focus on today. The photos coming round are of the activity workshops (rugby/basketball/dance) and the HLW.

*Pass the photos around the table

*Have a copy of goals sheet visible

*Give the children a Post-it note each and a pen

Before we get started we are going to ask you to imagine you are describing the Healthy Lifestyles Programme to some Year 5 children who are just about to start but don’t know what it’s all about. Can you come up with 3 words that you would use to describe the whole experience to other children?

There is a voice recorder in the room so we can write up notes about the things you say, please don’t worry about it being on. We will go round the room and each say our name, so let’s get started…..

What did you enjoy about the HeLP Programme? (one thing from each child) Probe reasons for why they enjoyed these things

Did you talk about HeLP at home? *Probe what was said and with whom and why they wanted to talk about these things*

What do you think your parents/family think of HeLP? *Probe events they*

*came to and if anything was said/discussed as a result*

Did you talk about HeLP at school e.g. in the playground or in other lessons? *Probe what was said and with whom and why they discussed these things*

What did you think about the drama and the other activities during the Healthy Lifestyles Week?

How was it different to what you usually do in school? *Probe why and the impact this had on them*

How easy was it to pick a character? *Draw out why they identified with their character*

Did working with your chosen character help you to set goals? *Probe reasons why*

How did you feel when you had a different actor playing your character in the year 6 activities? *Probe reasons for these feelings*

How did you find setting goals at home with your parents? *Probe reasons for their experiences. Probe if anything was discussed/said at this time*

What was it like trying to achieve your goals? (at home and at school). *Probe reasons for why it was difficult/easy*

Could you tell us anything you or your friends and family did to help you achieve your goals? (at home and at school). *Probe what they were and the impact it had*

Have you noticed any changes in yourself from being involved in the Programme? *Probe what they were. Make it clear to the children this can include their thoughts and attitudes as well as their behaviours*

Have you noticed any changes at home since the Programme? *Probe what they were. This can also include discussions and how they relate to their family as well*

Was there anything about the Programme you didn’t enjoy so much? *Probe reasons why*
